# Green Organizational Culture, Corporate Social Responsibility Implementation, and Food Safety

**DOI:** 10.3389/fpsyg.2020.585435

**Published:** 2020-11-06

**Authors:** Xiao Liu, Kuen-Lin Lin

**Affiliations:** ^1^College of Tourism, Beijing Union University, Beijing, China; ^2^Department of Business Administration, Cheng Shiu University, Kaohsiung, Taiwan

**Keywords:** green organizational culture, corporate social responsibility, food safety, Walmart, perspective

## Abstract

Food safety, ultimately, is a human-centered work. No matter how regulations are coercively released and implemented, the free will and behaviors of human actors (e.g., employees) lead to a real result in food safety. A real motivator of such free will and behaviors is organizational culture that stimulates meaningful organizational actions. Based on such rationale, this conceptual article sets to discuss the relationships between green organizational culture, corporate social responsibility implementation (hereafter CSR), and food safety. As organizational culture has been largely discussed in Management and Business literature, green organizational culture and its impacts on socially and environmentally friendly organizational behaviors, as well as public health outcomes like food safety, is wanted. With the clarification of the relationships between these three important constructs, theoretical implications for future research and practical implications for governance and policy-making are well generated.

## Introduction

Environmental challenges, including pollution and global warming, are rationalized and identified as the primary issue that makes organizations and individuals evaluate measures for preserving the environment. The environmental challenges led to the initiation of environmental issues with marketing and consumer perception on the quality of products (Clark et al., 2019). The green consumerism urges organizations to put more emphases on advertising and promoting eco-friendly products with message content such as recycling, healthy, reusable, recycling, and eco-friendly (Liu et al., 2016). To date, however, green consumerism touches on a broad but external scope like marketing and advertising, with less concerning about internal imperatives such as green culture and its influences. By definition, green culture is a collective belief toward an ecological, environment-friendly style of (co)production shared by most organizational members. This leads to a gap in the literature that even fewer have investigated in the interactions between internal and external imperatives concerning green consumerism and product quality, especially in the food sector. Nonetheless, strategic actions toward external stakeholders, such as the implementations for corporate social responsibilities (hereafter CSR implementation), need to be stimulated by internal drivers (e.g., culture) of the organizations. To fill up the gap, this article sets to conduct an in-depth discussion of (internal) green organizational culture’s influences on CSR of organizations in food industry, and food safety. The rationale is elaborated as follows.

Food safety is a result from organizational members’ collective beliefs and efforts in environment and stakeholder friendly co-creations. Both collective beliefs and collective actions are necessary to offer high-quality food products/services. Here in our analysis, a green culture represents the collective belief, and CSR implementations represent the collective efforts. Note that the term “CSR” is a very broad concept and a broad field of research too. As Sheehy (2015) noted, CSR can be conceptualized as a “field of study, a management practice and an approach to improving the dialogue concerning the social contribution of business…” From such statement, it is necessary to offer a clear definition for CSR before we proceed further. Mainly, we set to talk about the CSR from an action/activity perspective—that is, what does a company do for CSR. There are different views on the concept of the corporate social “responsibility,” such as CSR as a philosophy or CSR as an action contributing to an organization’s environmental, social, economic, and even political imperatives. While the former is more close to the concept of culture, we wish to take an action perspective of CSR and emphasize that the implemented CSR efforts/actions can be distinctive from, and be influenced by, organizational culture.

**FIGURE 1:**
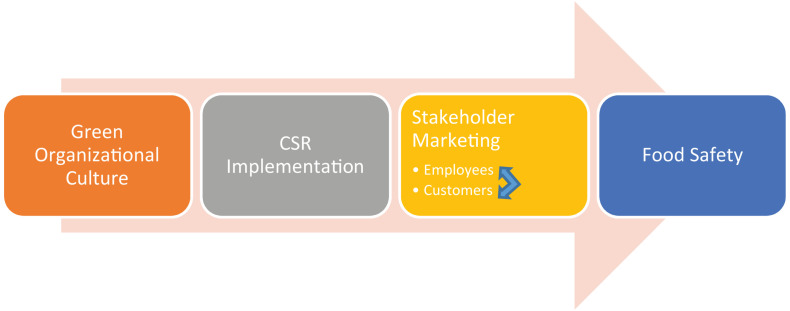
A tentative framework for conceptual analysis.

Thus, from an inside–outside interaction perspective, on the one hand, companies that operate under a green organizational culture recognize, analyze, solve issue/problems, and develop strategies that uniquely help the company to navigate through the environmental values (McCullough et al., 2016). In such sense, a green organizational culture reflects that the people and organizations need to have a cultural transformation for the collective awareness of their collective actions toward stakeholders and environments (Aliyu et al., 2015). From the market perspective, on the other hand, consumers’ choices of food to purchase depend on the marketing features of food enterprises, including word of mouth, which maybe be identified with the enterprise’s organizational attributes such as the green culture that stimulates its external, stakeholder-favorable behaviors such as the CSR implementation here, and we wish to clarify the impact of such implementation of CSR (which is thus tangible/visible/communicative to internal and external stakeholders) on food safety as an ultimate collective value generate. In sum, from both perspectives, it is important to elaborate on the collective attribute-action-value nexuses of green culture, CSR implementation, and food safety. Such integration also justifies the central view of the Stakeholder Theory (Freeman, 1984, 1999; Freeman et al., 2010), which relies heavily on integrating resource-based and market-based views (Phillips, 2003) on corporate’s attributes and behaviors in influencing (and being influenced by) multiple internal and external constituencies and entities. Based on the discussions above, this article may generate the following contributions. Firstly, it analyzes *why and how* important internal environmental climate (i.e., the green culture) can lead to environmental and public health favored results (i.e., food safety), through effective external environment actions of organizations (i.e., CSR). Secondly, the article discusses the abovementioned issues in a context of a nested, multilevel ecosystem that binds and bonds stakeholders, objects, processes, etc., in nature; the dynamics between green culture, CSR and food safety is embedded in different levels within an organization, which involves a diverse set of stakeholders (Liu et al., 2016). A lot have been said about how those issues are important individually, though, far less have tried to explicate their importance collectively and interdependently. Hence, we set to discuss a cross-level model to generate a broader thought of the phenomenon that might importantly occur at the intersection of green culture, CSR, and food safety in modern organizations. This also constitutes another contribution of this article—to unleash the complex interrelationships between the three main constructs, for which the phenomenological nature is a complex and multilevel system. Third, the article offered an interdisciplinary discussion of the issues. To facilitate our discussions of such topic and correspond the issue’s phenomenological natures, this article was positioned from an interdisciplinary perspective. Specifically, green culture is a social-psychologically shared climate among members (Social Psychology), CSR deals with a potential tension between cost (Economics) and good governance (Business Management), and personal and group motivation and conducts in food safety are greatly affected by psychological and ecological concerns (Psychology & Ecology, or Ecological Psychology). A conceptual framework can tentatively facilitate readers’ understanding of the core rationale of this article, as the following Figure 1.

## Green Organizational Culture

The terms for describing the concept of a green organizational culture have been diversified, including eco-friendly culture, environment-friendly culture, sustainability-oriented culture, etc., which is mainly based on extending organizational culture to a green oriented context. For instance, Norton et al. (2015) defined a pro-environmental organizational culture as they extended Schein’s (1990) organizational culture definition to meet the practical and perceptual criteria of working on business with the premise in environment conservation and protection. It is a widely accepted approach which we followed by defining green organizational culture here as a set of collectively shared beliefs, values, perspectives, norms, and even practices, which guides organizational members to behave properly toward the external environment during economic business processes.

Green organizational culture is one of the most debated topics by both the laymen and elite classes of people in society. The green culture concept is mostly concerned with realizing and obtaining the ecological balance (Mohezara et al., 2016). It involves both environment and people hence the need to carry out green culture since it promotes ecological development and sustainable economic growth based on politics, science and aesthetics (Galpin et al., 2015). Through globalization, different economies have shared the benefits of undertaking green tendencies and incorporating such practices in their organizational culture. Most of the organizations are restructuring their cultures to accommodate new factors on issues such as environmental ones, behavior, and attitude related to environmental problems (Firoz and Abinakad, 2016). Various scholars have identified the theory of reasoned action to establish the relationships between intentions, attitudes, and behavior based on the purchase of green products. A study conducted in Portugal and Brazil identified the influence of mediating variables in the course of purchasing green products. Some of the models used to describe the behavior and need for green purchase included quality and price (Fuentes and Fredriksson, 2016). However, other researches have faulted this finding claiming that price and quality are situational factors and not the chief elements.

The increased demand for green culture across the world has forced specialty stores, departmental stores, and shopping malls to stock up numerous products, both useful and useless. The entire practice has threatened the welfare of people and the ecological balance, with most industries becoming a source of different pollutions to the environment and against the people (Harrison, 2019). The environment is deeply affected by the production, consumption, and disposal of manufactured products. Nature is behaving unnaturally because of the excessive pollution with events such as heavy rains, floods, drought, and global warming. The natural calamities such as earthquakes, cyclones, tsunami, and other epidemics have become more frequent. Green culture is attempting to protect the welfare of consumers and the environment through production, consumption, and disposal of eco-friendly products.

The framework of the administrative culture plays a crucial role in ensuring successful cultural transformations. Every organizational culture has three levels, including values, assumptions, and artifacts, which reflect companies’ requirements and desires concerning their environmentally sustainable operations. Generally, green culture is the holistic internal marketing concept involving the production of materials, consumption, and disposal of both products and services. With the detrimental effects the environment is facing today, the production team, dealers, and consumers have become increasingly sensitive to developing and consuming green services and products. Green culture seeks to increase the production of pure products by conservation of energy, pure technology, preservation of the environment, and minimum natural resources in the production process. The use of natural foods in place of processed synthetic foods is the primary campaign in green culture, calling on people to ensure they start consuming natural foods because of the health factor (Bortolotti et al., 2015).

## The Green Organizational Culture and CSR

Corporate social responsibility is a set of self-stimulating and self-regulating organizational affairs that facilitate an organization’s reputational and realized capacity to be trusted and accounted by its stakeholders – and thus potentially lead to social values created to generate feedback for the organizations own value. CSRs in organizations encompass a series of facets, including sustainable sourcing, volunteering, ethical labor practices, community outreach, and environmental conservatism. In practices, CSR takes many forms to happen, such as donations for who needed, facilitation of community development, social problem re-solving, etc. Through the CSR programs, businesses not only create economic value but also strengthen communities and create healthy ecosystems. Partially similar with green organizational culture, CSR may also be stimulated when facing the blame for impending the green movement of businesses or when companies consider the sustainability goals (Tang et al., 2016).

As a driver, the green organizational culture is accountable for CSR, as CSR involves managing the social, environmental and economic risks in the process of decision-making (Firoz and Abinakad, 2016). The green organizational culture is made successful by a group of employees who understand the course in supporting environmental management. Hence, an organization with a strong green culture is equal to one that has a supporting workforce for engaging in CSR activities. Under the CSR, companies that bear a green culture thrive in the sustainability business while making strides in protecting the environment and attracting the most talented employees in the process. An organization that practices green culture not only attracts business but talent too. Companies today are gauged as per the CSR aspect with the people waiting to evaluate the activities the company engages in to help the society (Clark et al., 2019). A sustainable business will advocate for recycling of plastic waste, cleaning the water ways from plastic materials or providing solar panels for households that rely on fossil fuels for lighting. Cultural matrices are conditions that guide the daily lives of humans, integrating them with nature in a community. It is impossible to demystify a separation of the culture from the environment and its people. A green organizational culture will ensure all the practices of the business including the HR practices are in line with the green movement of environmental sustenance (Cordeiro and Tewari, 2015). The HR practices lead to a supply chain that observes the green mandate of the business culture while moderating the relationship of sustainability culture and overall business performance.

## Effects on Food Safety

Food safety begins from the production process to the packaging, from advertising and marketing to reselling in the local and international markets. Health awareness reflects individuals’ thoughts on fitness issues and their willingness to execute actions that will ensure their health is achieved (Liu et al., 2016). For example, nowadays consumers have developed an increasing need to undertake actions that will ensure their health remains intact by considering nutrition. A general belief that consumers possess is that organic food is healthier than inorganic or processed since it is chemical-free and rich in nutrients (Cordeiro and Tewari, 2015). Health consciousness is one of the major factors that result in predictions of constructive attitudes to food safety (Han et al., 2017). Consumption of food is associated with environmental issues, including pollution, water scarcity, and increased greenhouse gas emissions (Kolk, 2016). The consumption of beef, for instance, has a significant effect on the ecosystem since the production of proteins generates a substantial quantity of carbon dioxide. Pain, health problems, and death are some of the effects of consuming high amounts of microorganisms and residues. Food-borne illnesses result in losses and medical costs to the public health sector. It is imperative to adopt more maintainable food deeds with the greatest importance for facilitating conservation sustainability and public well-being. Not just for consumers as a pulling force of food safety, it is equally important for employees in organizations to be conscious in food safety. If the employees, who are generally perceived as “internal customers,” do not contribute to food safety, the effects from an organization’s food safety strategy and its customers’ food safety demands would be weakened, due to the missing efforts from the mediating employees. The psychology and purchase behavior of employees are affected by various factors, including the culture of the organizations and the actions taken under such culture. In such premise, CSR has become an instrument nurtured by the green organizational culture to influence employees’ values and commitment in food safety. Such values and commitment in food safety of employees can be seen or felt by customers and thus affect consumers’ purchase intentions and behaviors (Bortolotti et al., 2015). For example, in like manner, most organizations have ensured that green initiatives are delivered through corporate social responsibility (CSR) projects to entice buyers (Cordeiro and Tewari, 2015). Thus, the promotion of a company’s corporate social responsibility spirit and what has been done frequently by the employees can be saturated into the market through messages displayed on the company’s social media platforms.

Initially, engaging in CSR was a feeling-good concept for the organization, but today, it goes beyond that feels. In consideration of food safety, CSR can affect a corporation’s bottom line, particularly with the augmented difficulty of supply chains and the importation of yields and ingredients (Firoz and Abinakad, 2016). Social responsibility is directed to food safety in various ways, including snowballing market scope for significant concerns resulting in increased imported merchandises as components for processed nourishments. It leads to contradictory labor laws across countries and their global oversight, which requires vigilance in regards to the safety and rights of people (Clark et al., 2019). The social media impact that s growing significantly has forced consumers to ask food companies to engage in CSR practices. Food companies need to execute CSR practices and provide a safe environment for their employees. Unsafe working environments are likely to result in employees working less to provide a safe processing environment for its customers (Elder and Dauvergne, 2017). Other effects of the unsafe working environment include increased employee turnover rate, presence of increased bloodborne pathogens, heightened employee accidents while at work due to loss of oversight or attention, and other possible safety events. Most of these circumstances directly influence the safety of merchandises, causing an uproar on social media and the company’s website.

CSR operations not only show what a company stands for but also show what it stands up for and the efforts it is willing to put. Most of the food processing companies rely on sugars, and generally, the overly used type of sugar is the GMO-sugar because it is readily available. Companies that sought non-GMO sugar have to find other suppliers who, in most cases, are further, and shipping takes longer for the supplies to arrive at the factories (Havinga et al., 2015). Social responsibility means taking the lead in social justice and ensuring that a company will take the risk for the sake of the people. Such companies are mindful of their profits, but above all, they are mindful of their consumers, which is the highest form of CSR.

In the era of social media where everything is reported directly, it is only fair for companies to adopt an accurate measure of dealing with their CSR activities. Consumers have become more aware of products that mean well for them and those that do not. Companies that have made negative comments to the clients have faced severe consequences, including campaigns to turn customers against purchasing from their stalls. A research conducted in 2018 acknowledged that most consumers who purchased food products were guided by the company’s social and environmental activities. If a product contributed positively toward an issue a fraction of society cared for, the consumers would spread the word to their peers, parents, and workmates.

Consumers are becoming more vigilant and educated as they realize how the process of food production leads to environmental damage and causes human suffering. Pepsi and Coca Cola were put on the spotlight by Oxfam, which reported issues that communities growing sugar crops endured. Such companies purchased their land without respect for people’s cultural rights. The two companies announced that they would improve their purchase processes. They had to announce the environmental concerns raised about the lands they were acquiring from communities. Besides environmental and health issues, companies need to be careful about other social issues that affect people across the world, such as women’s rights. Various companies have been forced to make public announcements to pledge their support for women and align with their interests.

CSR is no longer adjacent but incorporated into the core business practices because of the paramount role in the progress of the company and its growth. CSR efforts are meant to assure the type of food, the process of production, and its safety at a time when most of the people are more aware of their health. CSR is part of today’s corporate growth strategy because only the companies that are putting effort to ensure the health and wellness of the people will attract the best talent, build stronger partnerships with employees, manage risks of supply chains and allow transparent conversations with investors and key players in the industry. Companies have paddled back to the suppliers and engaged them for more lessons on how to minimize production costs and make their products more organic. Companies are building platforms for transparency, honesty, and traceability, ensuring sustainable and ethical practices along the entire supply chain.

Correlating CSR positively with competitively is an imperative factor for the consumer perspective because it plays a significant role in mediating between the two to result in increased demand for products (Wei and Huang, 2017). Consumer behavior and perception are concepts that have been studied widely by various scholars to determine the effect of CSR and purchase intention. CSR perception is extensively misunderstood across the world; consumers take it as a company’s commitment toward bearing social responsibility (Aliyu et al., 2015). This means that businesses need to pay keen attention to the interests of the immediate community it seeks to serve. With the heightening interest in protecting the environment, consumers link CSR with the mindfulness of a company to protect the environment responsibly. The literature review insists that consumers have different attitudes when exposed to different sources about the same. What stands out in the world today is the need to take care of the environment and manufacture food products in tandem with the current health needs.

A company that seeks to deliver these requirements makes it possible for the consumers to resonate with its products. The attitude and perception of consumers toward the products related to the CSR activities that portray care to the environment and health of its consumers. In contrast, negative information about unethical actions taken by a company sends the wrong message to the consumers, affecting their attitude toward the brand (Clark et al., 2019). Companies are judged, and the merits of their CSR weighed by the public on social media. If the activities are seen as a way of taking advantage of other companies, public criticisms for irresponsible behavior hurts the company.

The CSR of companies across the world is a critical factor that calls for strategic planning. In the 80s, when CSR was introduced in companies, it only involved supplementary activities that would not affect its profits. Currently, CSR is a factor that determines the growth of a company hence calling on the management to choose CSR activities that impact the company positively. The environmental considerations have manifested in ways such as commitments, proclamations, and promises to improve the livelihood of the people it serves. Some of the most prominent activities include providing eco-friendly packaging material, adopting the most sustainable manufacturing modes, and processing. Most companies have initiated a recycling program that allows consumers not to litter but return the packaging material to the company (Liu et al., 2016). The health of every individual is dependent on their surroundings.

Food safety is paramount in the current world because of the increased cases of illnesses associated with manufactured and processed inorganic foods. The public may not be aware of the communicated CSR activities by the company. The retailers and manufacturers have started communicating CSR activities on social media to reach as many consumers as possible (Tang et al., 2016). Retailers distributing food products need to provide candid information to the consumers about the food source, the processing plants, and the entire supply chain. Most companies display this information because of the need to assure the consumers of the quality and safety of foods (Cordeiro and Tewari, 2015). The impact of CSR is felt by companies that are dedicated to supplying foodstuff to the consumers. Most of these retailers have committed continuously toward establishing a framework to help the farmers and help their employees to advance their careers.

## Conclusion and Theoretical Implications

Social consciousness is one of the factors determining the growth of the company (Yang and Gong, 2020) as companies seek to streamline their online presence, inventory, and distribution management practices, a dedicated framework toward a workable and active CSR. Doing so, economic considerations like cutting costs are still paramount for some company but for food retailers, some have had to acquire plantations to assure the public of their quality and ensure it is constant. Companies are publicizing their CSR activities since they stand a chance to gain support from various NGOs and administrations who find CSR to be a way of improving environmental and social conditions in places that were not attended due to financial capacity (Nouaimeh et al., 2018). When companies use CSR to publicize their efforts to facilitate goodwill to the country, they not only win the support from NGOs to increase their legitimacy but also find public endorsement.

Consumer’s perception, behavior, and intention of buying are positively affected by the CSR activities of a company. Most of the consumers are concerned about food safety. Companies that outsource their products from such markets have to contend with unsatisfied and disgruntled customers who assume that the foods are unsafe for their consumption. Consumers are apprehending companies to be transparent about their source of products, the standards of growing the food, packaging, and distribution. As explained above, CSR grounded on a sound green organizational culture helps companies gain employees supports in action, as well as a broader market from consumers caring about food safety. From this article, one can note that consumers are more assured of food safety when companies have green organizational culture that can lead to CSR activities that engage employee’ commitment and actions toward food safety. The internal green culture affects the buying culture of consumers through the CSR activities and engaged employees.

Our discussions generate several theoretical implications for future research reference. First, as a fresh perspective for the interlink between the green culture, CSR, and food safety was proposed, empirical studies can test theoretical models incorporating a part or all of the interrelationships between the major constructs abovementioned. Second, the discussions distinguished and shed light of the potential interactions of internal employees and external customers, on the basis of an organization’s green culture and stimulated CSR. Future studies of environmental issues such as the food safety here should also consider the two-folded and interactive influences on and between the two important stakeholder groups. Third, future studies are also encouraged to explore the outcome of CSR that was not in its original purpose. Most studies examine CSR’s outcomes that seemed directly and reasonably related (performance, reputation, satisfaction, etc.) This article used food safety to (hopefully) imply the potential of widening the exploration of CSR’s influences on outcomes in different phenomenological and research areas. Last but not least, further elaborations/examinations with dimensionalized constructs discussed in this article may further create more opportunities for study. For example, can non-food-related CSR (e.g., ecological or environmental CSR) also increase internal employees’ awareness of food safety? What are the comparative effects of food-related versus non-related CSR? Similarly, dimensionalizing the green culture may generate more such opportunities and research questions to explore.

## Data Availability Statement

The original contributions presented in the study are included in the article/supplementary material, further inquiries can be directed to the corresponding author.

## Author Contributions

XL wrote the original manuscript. K-LL revised and edited the drafts. Both authors contributed to the article and approved the submitted version.

## Conflict of Interest

The authors declare that the research was conducted in the absence of any commercial or financial relationships that could be construed as a potential conflict of interest.
